# Novel Approach for a Controlled Delivery of Essential Oils during Long-Term Maize Storage: Clove Bud and Pennyroyal Oils Efficacy to Control *Sitophilus zeamais*, Reducing Grain Damage and Post-Harvest Losses

**DOI:** 10.3390/insects14040366

**Published:** 2023-04-07

**Authors:** Pedro A. S. Sousa, Joana Neto, Joana V. Barbosa, Joana Peres, Ana Magro, Graça Barros, José M. Sousa, Fernão D. Magalhães, António Mexia, Ana A. R. M. Aguiar, Margarida M. S. M. Bastos

**Affiliations:** 1 GreenUPorto—Sustainable Agrifood Production Research Centre/Inov4Agro, Faculty of Sciences, University of Porto, Rua da Agrária 747, 4485-646 Vairão, Portugal; up201200288@fc.up.pt (P.A.S.S.);; 2 LEPABE—Laboratory for Process Engineering, Environment, Biotechnology and Energy, Faculty of Engineering, University of Porto, Rua Dr. Roberto Frias, 4200-465 Porto, Portugal; 3 ALiCE—Associate Laboratory in Chemical Engineering, Faculty of Engineering, University of Porto, Rua Dr. Roberto Frias, 4200-465 Porto, Portugal; 4 Instituto Superior de Agronomia, Universidade de Lisboa, Tapada da Ajuda, 1349-017 Lisboa, Portugal; 5 Departamento de Química, Escola de Ciências da Vida e do Ambiente, Universidade de Trás-os-Montes e Alto Douro, Quinta de Prados, 5000-801 Vila Real, Portugal; 6 LEAF—Linking Landscape, Environment, Agriculture and Food, School of Agriculture, University of Lisbon, Tapada da Ajuda, 1349-017 Lisboa, Portugal

**Keywords:** eugenol, food security, insect control, maize weevil, *Mentha pulegium*, pulegone, *Syzygium aromaticum*

## Abstract

**Simple Summary:**

*Sitophilus zeamais* (Motschulsky) is one of the most destructive pests of stored maize grains worldwide. Synthetical chemical insecticides are applied for its control, but these can cause the development of resistant populations and have environmental implications. Essential oils can be a viable alternative to synthetic chemical insecticides, but their long-term effectiveness is still up for debate. The aim of this work was to evaluate the effectivity of Clove bud and Pennyroyal essential oils for long-term (twenty weeks) protection of maize, delivered with the aid of an innovative macro-encapsulation device. The blend of both compounds reduced losses by more than 45%, diminishing the survivability of *S. zeamais* by over 90%. This work demonstrates the potential application of this technology and solutions on the control of *S. zeamais*, describing and evaluating their effects on *S. zeamais* populations and their relation to the damages and losses of maize grains.

**Abstract:**

Maize grains represent a significant contribution for assuring food safety all over the globe. *Sitophilus zeamais* (Motschulsky) (Coleoptera: Curculionidae), also known as the maize weevil, is one of the most destructive pests in stored maize, causing qualitative and quantitative losses. To control *S. zeamais* populations in maize storage sites, synthetical chemical insecticides are applied. However, these are often used wastefully, have environmental implications, and can induce the development of resistant populations. In this work, the insecticidal and grain protecting efficacy of an innovative macro-capsule delivery device, loaded with essential oils from Clove bud and Pennyroyal, as well as their combined solutions, was tested against naturally *S. zeamais*-infested maize grains. The blend of both compounds incorporated in a controlled release device reduced losses by more than 45% over a long storage period of twenty weeks, diminishing the survivability of maize weevils by over 90%. The usage of the blend at a concentration of $370\;\mu L\cdot L_{\mathrm{air}}^{-1}$ with an antioxidant showed the best results, however, by halving the concentration $\left(185\;\mu L\cdot L_{\mathrm{air}}^{-1}\right)$, a significant control *of S. zeamais* populations was still achieved.

## 1. Introduction

For the last two decades, the world’s total population has seen a substantial increase, recently reaching the projected number of 8 billion people by November 2022, adding 1 billion people since 2010 and 2 billion since 1998, marking a significant milestone in the world’s population [1]. Concurrent with the populational growth, worries about food availability and security have likewise increased, and are now aggravated by the effects of the recent pandemic and the conflict between two major worldwide agricultural players [2]. Cereal grains are one of the most important food commodities, with major relevance in the global economy. Particularly, maize represents a significant contribution to food safety and its consumption has been increasing each year, mainly due to its versatile nature as a product that can be consumed as a whole for human food, while also being used for livestock feed [3]. In Portugal, maize is the cereal with the highest quota in landmass usage, encompassing over 100,000 ha of cultivated area [4].

Storage of harvested maize grains is a critical step in securing food safety, all while warranting the availability of seeds for the next planting season and mitigating eventual fluctuations in market availability [5]. It is also during post-harvest when the majority of losses occur; insects are the main culprit, accounting for up to 50% of the damages during this stage [6]. *Sitophilus zeamais* (Motschulsky) (Coleoptera: Curculionidae), also known as the maize weevil, is one of the most destructive pests in stored maize worldwide, causing qualitative and quantitative losses of maize grains [7,8,9]. *S. zeamais* is an internal feeder, with pre-adult stages developing inside the grains. Larvae consume the contents inside the grains during development with adults continuing the spread of damages throughout its life cycle, both reducing the kernels to a powdery form [10,11]. Directly, damages inflicted by *S. zeamais* by its feeding and development make the market value of maize decline, as well as reduce germination rates, seed weight, and the nutritional value of the grains, which are all factors that exacerbate food safety issues [12,13,14]. These damages are further aggravated by the increase of temperature and moisture content caused by proliferation of the pest, favouring the establishment and growth of microorganisms, such as phytopathogenic fungi, that synthetize harmful mycotoxins [15,16].

To control *S. zeamais* populations in maize storage sites, synthetical chemical insecticides are applied. Phosphine utilization via fumigant application is common practice, as well as the employment of organophosphorus and pyrethroid compounds as grain protectants [17]. Although effective, the usage of these compounds can have important drawbacks: for instance, mishandled applications of phosphine through typical fumigation methods can be extensively wasteful [18], with reports of selective resistant populations of *S. zeamais* to pyrethroids, organophosphates, and phosphine in South America [19]. Likewise, the usage of synthetical agrochemicals can be hazardous for those handling them, especially when appropriate equipment and knowledge are not available, as is often the case in under-developed countries. Furthermore, current European goals to reduce the use of synthetic agrochemicals [20] with restrictions to the usage of several neonicotinoid compounds may result in the repetitive application of a narrower range of insecticides [21], one of the main factors to enable the onset of resistant insect populations [22]. Thus, there is a need to research safe, effective, and sustainable compounds and methods that can be introduced into integrated pest management practices for the protection of stored maize grains.

Essential oils (EOs) are plant-derived compounds with known insecticidal properties that have been widely studied in the last decades as safer alternatives to conventional synthetic insecticides. These products have been emerging as a preponderant biorational alternative for the integrated management of stored insect pests of maize, with promising results on their efficacy against *S. zeamais*, both as a fumigant as well as a contact-based insecticide [23,24,25,26]. EOs are synthesized in secondary metabolic pathways as a mechanism to protect the plant against direct and indirect damages by biotic factors [27]. Since its synthesis and accumulation is associated with many secretory structures, EOs can be extracted from a plethora of plant parts, such as buds, flower petals, stem rhytidome, leaves, seeds, roots, resins, and fruit peels [28]. As a crude extract, EOs are complex in their nature, comprising a great number of polar and non-polar molecules. Often, two to three components are predominant and determine the bioactivity of the oil [29,30].

Eugenol (C_10_H_12_O_2_) is a phenolic aromatic substance known to be present predominantly (75–85%) in the EOs of *Syzygium aromaticum* (L.) buds and leaves, and cinnamon leaves (*Cinnamomum zeylanicum* (Blume), *Cinnamomum cassia* (Blume), and *Cinnamomum verum* (J. Presl) [31], although it can also be produced synthetically by the allylation of guaiacol with allyl chloride [32]. The usage of eugenol as a biopesticide is well recognized, with shown repellency, contact, and fumigation toxicity against a wide range of insects, including aphids, armyworms, beetles, cutworms, grasshoppers, loopers, mites, and weevils, such as *S. zeamais* [33,34,35,36,37,38]. Eugenol has also been shown to be able to hyperactivate insects’ metabolism [39] and increase food intake, a characteristic that promotes its high synergistic potential [40,41,42,43].

Pulegone (C_10_H_16_O) is a monoterpene ketone found mainly in EOs extracted from plant species belonging to the Lamiaceae family. From these, it can consistently be extracted in higher quantities (75–85%) from *Mentha pulegium* (L.), commonly known as pennyroyal [44,45]. Pulegone potential as a bioinsecticide relies on its acute toxicity. This monoterpene has been reported on various insects to be metabolized into menthofuran when consumed, which is a highly toxic organic compound that seems to follow an oxidative pathway yielded by the cytochrome P450 [46,47,48]. Herrera et al. [49] has similarly reported the same occurrence in *S. zeamais*, and several other authors have observed acute toxic effects against the same pest [18,50,51,52]. Synergistic effects have also been observed for pulegone, including in combination with eugenol, but it seems to be highly reliant on its compatibility with other compounds in the mixture [40,49,53].

Eugenol and pulegone are generally regarded as safe towards mammals and in lower dosages, they may even exert beneficial effects [54]. Despite the great potential of these substances, there are some drawbacks that limit their widespread usage as crop protectants against agricultural pests and diseases. EOs, including eugenol and pulegone, have poor solubility in water and are susceptible to oxidation and degradation, all characteristics that severely hamper their persistence and applicability, especially for longer periods of storage [34,55,56,57]. As such, repeated applications of EOs may be required in order to maintain their insecticidal effects. This hampers their practical usage and restrains their economic feasibility in real-world applications. To overcome these disadvantages, the delivery of EOs through encapsulation techniques for a controlled long-term release has been one of the key areas of research in the last decades for this topic. To name a few, EOs can be delivered through encapsulation in polymeric matrixes, micro/nanoencapsulation in polymer-based particles and micelles, nanoemulsions and microemulsions, and cyclodextrins [58,59]. Post-harvest, other techniques can also be applied for the delivery of EOs, such as the impregnation of polyethylene films in food packaging and preservation, including low density polyethylene films and plasticized delivery matrices for the protection of maize grains against *S. zeamais* [18,49,60,61,62].

Previous studies tackled the effectivity of these compounds in combination with controlled release devices and matrices to control the maize weevil. Despite the general consensus about the potential application of EOs for the control of *S. zeamais* during maize grain storage, their effectivity during long periods of time in a larger scale setting are still up for debate. By utilizing an innovative macroencapsulation device for the controlled release of these substances in a semi-practical setting, mimicking the conditions inside a maize grain storage silo, the aims of this study are to: (i) observe and evaluate the effectivity of Clove bud and *Mentha pulegium* EOs, as well as their roles in combination in controlling *S. zeamais* over the course of approximately five months; (ii) understand the potential applicability of this long-term maize storage technology through an evaluation of damages and quantification of losses throughout the whole experiment; and (iii) identify the possible influence of these compounds in the amount and type of losses produced per maize weevil.

## 2. Materials and Methods

### 2.1. Insects and Maize Preparation

Commercial grade dry maize kernels were utilized in these experiments and purchased from CARNEIRO CAMPOS & Ca S. A., Porto, Portugal. Since the purchased maize reached the supplier directly from the producer, grains were already naturally infested thoroughly with pre-adult and adult forms of *S. zeamais* throughout the whole silo bag at the time of purchase and thus, no artificial infestation was necessary. The maize was therefore used as is for the experiments, only being processed through manual sieving to remove any residual maize powder and other smaller debris and contaminants.

After the sieving process, the maize was thoroughly mixed, and random samples were collected and evenly distributed in a volume of 2.5 L in polyethylene containers (109 cm × 109 cm × 230 cm, nominal volume of 2 L, ref VWR 215-3248, Corning Incorporated, New York, NY, USA) closed with a HDPE (High density polyethylene) cap with a seal, each filled with 1.5 kg of maize, corresponding to ≈70% of the internal container volume.

The experiments were conducted inside a dark climatized room at 26 ± 2 °C. Ten replicates were prepared for each treatment, five of which were collected after 10 weeks of storage (OBS1) and the remaining five after 20 weeks of storage, at the end of the experiment (OBS2).

### 2.2. Chemical Products and Delivery System

**Figure 1:**
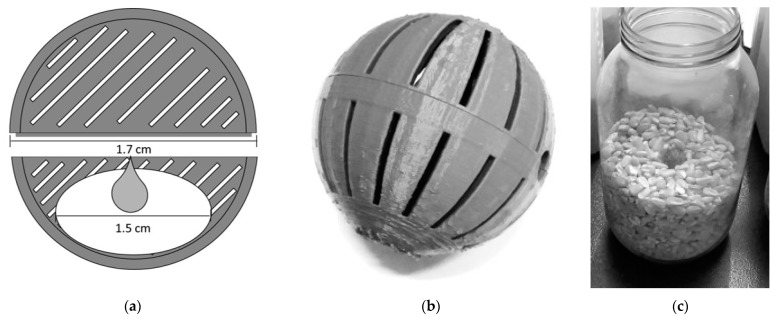
Illustration of the device technology utilized in the experiments: (**a**) Longitudinal sectioned diagram of a device, representing its dimensions and positioning of the compounds to be tested; (**b**) The 3D-printed complete device after assembly; (**c**) Position of the device inside a 2 L half-filled container (transparent glass flask used for demonstration purposes).

Each treatment consisted of using a 3D-printed biodegradable device made of a polylactic acid polymer for the controlled release of various solutions prepared from two selected essential oils in various concentrations. These devices are spherical (1.7 cm in diameter), made from two symmetrical half spheres, each with stripped openings to allow for the release of volatiles, and snaped together by an edge (Figure 1a,b). A small cotton disk (1.5 cm in diameter) embedded with the various testing compounds was placed inside each device. At the start of the experiment, each device was placed centrally halfway inside the maize filled containers (Figure 1c).

The choice for the base concentration of the compounds to be tested (i.e., the proportion of liquid compound to the volume of air in an empty container) was based on previous work that showed a toxic effect of *M. pulegium* EO in *S. zeamais* adults and progeny at a concentration as low as $20\;\mu L\cdot L_{\mathrm{air}}^{-1}$ for a period of 7 days [63,64]. Since this study focuses on the long-term protection of maize, the decision was to use a $60\;\mu L\cdot L_{\mathrm{air}}^{-1}$ baseline concentration. For simplification purposes, the EOs from *M. pulegium* and Clove bud will be referred to during the rest of this paper by their active compounds, pulegone and eugenol, respectively. In total, eight solutions and a Blank control (B) (only maize grains) were prepared for the experiment:

- Two pulegone-based solutions: (R)-(+)-Pulegone at $60\;\mu L\cdot L_{\mathrm{air}}^{-1}$ (P60) and $185\;\mu L\cdot L_{\mathrm{air}}^{-1}$ (P185);
- Two eugenol-based solutions: Clove bud oil at $60\;\mu L\cdot L_{\mathrm{air}}^{-1}$ with Adjuvant (E60 + Adj) and without Adjuvant (E60). Adjuvant solution proportions: 30% Clove bud oil, 50% Dipropylene glycol, 5% Brij^®^ L23, and 15% water);
- Three solutions with a mixture (1:1) of eugenol and pulegone, at three concentrations: $164\;\mu L\cdot L_{\mathrm{air}}^{-1}$ (EP164), $185\;\mu L\cdot L_{\mathrm{air}}^{-1}$ (EP185), and $370\;\mu L\cdot L_{\mathrm{air}}^{-1}$ (EP370);
- A solution EP370 with the antioxidant Ascorbyl Palmitate (EP370 + AO).

Clove bud oil (eugenol ≥ 85%), Pennyroyal oil (as (R)-(+)-pulegone, 85% technical grade), Dipropylene glycol, Brij^®^ L23, and Ascorbyl palmitate were all purchased from Aldrich^®^ (Steinheim, Germany).

### 2.3. Sampling Procedure and Data Collection

The sampling procedure followed a sequential order, enabling collection of different samples for the posterior analysis of insect population and mortality, the assessment of damage in maize grains, and the quantification of losses.

Sieving drums were utilized in order to separate the insects, maize powder, and maize grains in each sample. Each sieving drum was comprised of two levels of square-holed sieving matrixes, sieving drum mesh number 10 (2000 μm aperture), and sieving drum mesh number 20 (841 μm aperture). During each sieving process, there were collected per container:

1. 200 g of maize kernels, randomly sampled from sieving drum mesh number 10. These samples were then stored inside plastic bags and kept at a temperature of −20 °C until a subsequent prospection of damages;
2. The *S. zeamais* adults from sieving drum mesh number 20;
3. Maize powder from the bottom of the sieving drum.

**Figure 2:**
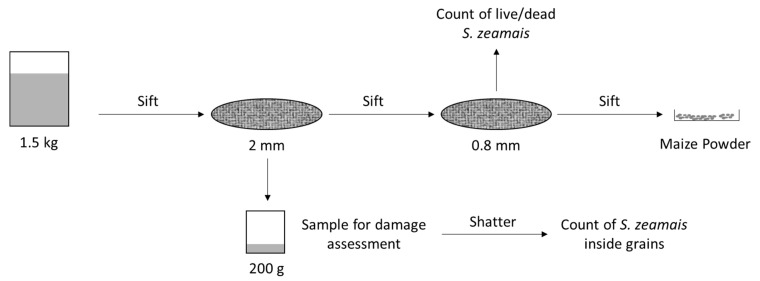
Schematic representation of the sampling process and collection.

Using the 200 g samples collected in the sieve, grains were separated according to the presence/absence of damage signs that could be attributed to activity of *S. zeamais*, and they were accordingly counted and weighted. Additionally, after the damage assessment was completed, grains with observable damages were individually and manually shattered to reveal eventual *S. zeamais* adults that could be inside the grains and thus were not observable in the initial assessment of live and/or dead adults. Since these grains were previously stored at −20 °C, the insects that were found inside these grains were only accounted for as the total number of *S. zeamais*, and not for the evaluation of mortality (Figure 2).

Collected *S. zeamais* adults were identified and divided between the ones that were showing activity and those that seemingly did not show any movement. Individuals that were showing movement were accounted for as live adults whereas the paralyzed ones were placed in a flat aluminum tray and observed for any sign of movement for 30 s while also being gently probed with a soft paintbrush. After this period, insects that did not show any activity were considered dead.

### 2.4. Population Dynamics and Damage Assessment

The assessment, estimation, and characterization of the *S. zeamais* (*Sz*) population were determined independently for every container, by counting the collected adult *S. zeamais*. The number of dead and alive adults accounted for during the sieving process were used for the estimation of mortality using the following formula:

(1) $$\mathrm{Mortality}\;\left(\%\right)=\frac{\mathrm{Number}\;\mathrm{of}\;\mathrm{Live}\;Sz}{\mathrm{Number}\;\mathrm{of}\;\mathrm{Live}\;Sz+\mathrm{Number}\;\mathrm{of}\;\mathrm{Dead}\;Sz}\times 100$$

As previously mentioned, *S. zeamais* adults found inside the maize kernels in the follow-up process were only accounted for as the total number of *S. zeamais*. Since these were prospected from a sample of 200 g, the number of *S. zeamais* found inside the grains were proportionally corrected for the estimated number of *S. zeamais* adults that seemingly were inside grains in the whole container:

(2) $$Sz\;\mathrm{inside}\;\mathrm{grains}\;(\mathrm{container})=\frac{Sz\;\mathrm{inside}\;\mathrm{grains}\times \mathrm{Total}\;\mathrm{maize}\;\mathrm{mass}\;(g)}{200\;g}$$

The total number of *S. zeamais* in each container was thus calculated using the estimated number of *S. zeamais* inside the grains in the container, adding the number of collected live and dead adults:

(3) Total number of Sz =Sz inside grains + Sz Live +  Sz Dead

**Figure 3:**
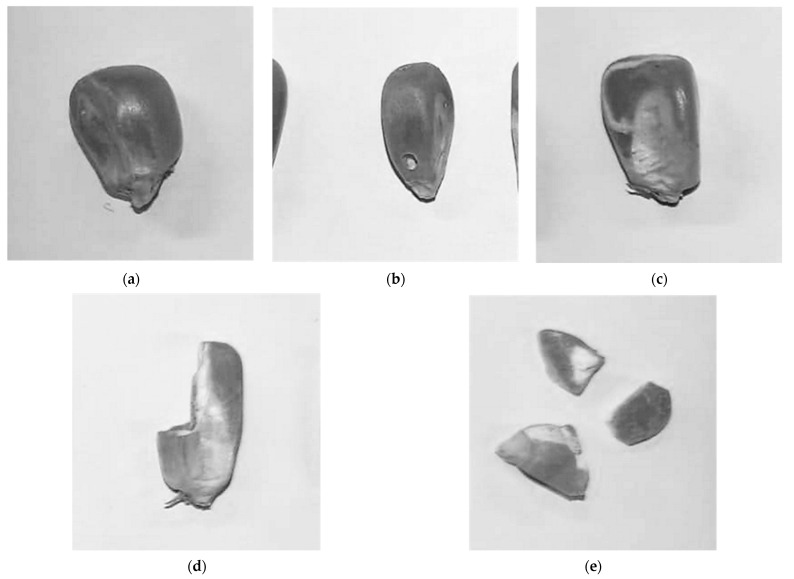
Types of damages in maize grains identified during sample collection: (**a**) Healthy Grain; (**b**) Perforated Grain (PerfG); (**c**) Grain with Galleries (GGall); (**d**) Chipped Grain (ChipG); (**e**) Grain Fragment (GFrag).

To better understand if the tested compounds could induce production of different types of damages in the grain by the insect, a grain damage index was created by describing the type of damage observed and grouping each grain and/or fragment of grain in different categories (Figure 3). Thus, the maize kernels were categorized accordingly:

1. Healthy grain—A grain was considered healthy when no sign of damage was observed (Figure 3a);
2. Perforated grain (PerfG)—A grain was considered perforated when a clear and round pierced hole was observed on the kernel surface, projecting to the inside of the kernel body (Figure 3b);
3. Grain with galleries (GGall)—A grain where at least one larva mine was observable, without any exit hole (Figure 3c);
4. Chipped grain (ChipG)—The grain body visually lacked a significant portion, but it remained at a size equal or greater than 50% of the average size of the measured healthy grains (Figure 3d);
5. Grain fragment (GFrag)—The grain’s size was less than half of the average size of the measured healthy grains (Figure 3e).

Assessment of losses was made using the weight of maize powder collected during the sieving process together with the count and weight method (*WGL*), as described by FAO [65] as per Harris and Lindblad [66] and Boxall [67]:

(4) $$WGL\;\left(\%\right)=\frac{U\times Nd-D\times Nu}{U\left(Nd+Nu\right)}\times 100$$

This method utilizes the differences between the number and weight of damaged grains relatively to healthy grains, where *WGL* means weight grain loss, *U* means the weight of undamaged grains, *Nu* means the number of undamaged grains, *D* means the weight of damaged grains, and *Nd* means the number of damaged grains.

Perforated grains (PerfG) were the only type of grain damages classified as damaged grains (*D* and *Nd*) for the assessment of losses through the count and weight method. Although Grains with galleries (GGall) could also be considered for this assessment, it was noted that at this stage of damage, there was no loss of weight in the grain when compared with healthy grains. PerfG were the only type of grains with damages that could be unequivocally attributed to the activity of *S. zeamais*, which influenced the weight of such grains. Thus, these were considered as the damaged grains for the assessment of losses through the count and weight method.

To determine which sampled group of *S. zeamais* (total number of *S. zeamais* or number of *S. zeamais* alive) can better correlate with the types of damages present inside each container, as well as understand what type of damages are specifically caused by the activity of the maize weevil, a Pearson coefficient was computed between both the total number of *S. zeamais* and the number of alive *S. zeamais*, with relation to the amount of maize powder, *WGL*, and number of PerfG, ChipG, GGall, and GFrag.

### 2.5. Statistical Analysis

After checking normality, homogeneity of variances, and sphericity assumptions, pair-wise comparisons were made. One-way ANOVA with a post-hoc Tukey HSD test was performed independently for each sampling period (OBS1 and OBS2) to assess differences between treatments for the total number of *S. zeamais*, number of alive *S. zeamais*, *S. zeamais* mortality (%), maize powder (g·kg^−1^), *WGL*, number of PerfG, average weight of PerfG (AWPerfG), and number of GGall. Additionally, comparisons between treatments for the same variables relative to the blank control were made using a repeated measures one-way ANOVA with a post-hoc Tukey HSD test. The correlation of damages with the sampled groups of *S. zeamais* was assessed with Pearson’s analysis.

To understand the effects that eugenol and pulegone could have on quality and to infer the quantity of damages produced per insect, a linear regression model was computed for blank control, E60, E60 + Adj, and P60. The slope of the line was analyzed for the purpose of establishing a relation between the total number of *S. zeamais* and maize powder, *WGL*, number of grains with perforations (PerfG), and number of grains with galleries (GGall). 

All data analysis was conducted using *IBM^®^ SPSS Statistics 26.0*.

## 3. Results

### 3.1. Population Dynamics

The total number of *S. zeamais*, the number of alive *S. zeamais*, and the mortality were all significantly affected by storage time (*f*_0_ = 12.720, *p* < 0.01; *f*_0_ = 12.535, *p* < 0.01; and *f*_0_ = 65.905, *p* < 0.01, respectively). Additionally, time had a significant effect on the treatments applied for the same respective variables (*f*_0_ = 3.785, *p* < 0.01; *f*_0_ = 7.454, *p* < 0.01; and *f*_0_ = 13.302, *p* < 0.01, respectively). At the first observation (OBS1, 10 weeks of storage), the total number of *S. zeamais* was the highest on P60, P185, and EP370. However, apart from P60, treatments P185 and EP370, together with EP164 and EP370 + AO, had the lowest number of alive *S. zeamais*, with the highest mortality rates between all tested compounds and blank controls. At the second observation (OBS2, 20 weeks of storage), P60 and EP164 had the highest population densities of *S. zeamais* between all tested compounds and blank controls. These same treatments also had the lowest mortality in this period and the highest number of alive *S. zeamais* (Table 1).

Relative to the blank control, P60 and EP164 had a significantly higher density of *S. zeamais*; EP370, EP370 + AO, and EP185 had a significantly lower number of alive *S. zeamais*, reducing the number of adult insects by 97%, 94%, and 81%, respectively. As for the observed mortality, relative to the blank control, P185, EP164, EP185, EP370, and EP370 + AO showed significantly higher mortality. The data also suggests that, at the same concentration and relative to the blank control, eugenol (E60) had a significantly lower total number of *S. zeamais* than pulegone (P60), despite the observed mortality and the number of alive *S. zeamais* not being significantly different between these two treatments. There was no significant difference in adding the Adj to eugenol, nor adding the AO to EP370 in all the tested variables. When increasing the concentration of pulegone (P60 vs. P185), there was no significant difference in the total number of *S. zeamais*, though it was noted that the increase in concentration led to significantly higher mortality and a significantly lower number of alive *S. zeamais* at OBS1, relative to the blank control. In both observations, relative to the blank control, there was no significant difference in adding eugenol (EP185) together with pulegone (P185). For all the tested variables, at OBS2, relative to the blank control, it was also noted that an increase in concentration in the mixture only produced significant differences when increasing from EP164 to EP370; no significant differences relative to blank control were observed from EP164 to EP185, as well as from EP185 to EP370 for the total number of *S. zeamais*, mortality, and number of alive *S. zeamais* (Table 1).

### 3.2. Evaluation of Grain Damages

From the two sampled groups of maize weevils, the total number of *S. zeamais* was the variable with the strongest correlation with the observed damages: this variable was positively and significatively correlated with the *WGL* (*r*(88) = 0.688, *p* < 0.01), PerfG (*r*(88) = 0.686, *p* < 0.01), maize powder (*r*(88) = 0.361, *p* < 0.01), and GGall (*r*(88) = 0.356, *p* < 0.01). Since the number of chipped grains and the number of grain fragments (GFrag) were not significantly correlated with neither of the sampled groups (*p* > 0.05), these variables were thus excluded from further analysis of damages in regard to differences between the tested compounds (Table 2).

As for the damage analysis, EP370 + AO had a significantly higher average weight in perforated grains when compared with the blank control (B) at the second observation (OBS2). There was no significant difference in utilizing Adj in the E60 treatment. There was also no significant difference between eugenol and pulegone at the same concentration (E60 and P60), nor in adding eugenol to pulegone in a mixture (P185 and EP185). There was also no significant difference in increasing the concentration of the mixtures (EP164, EP185, and EP370). Despite not showing a significant difference, every treatment with pulegone in the formulation reduced the number of grains with galleries relative to the blank control. Time had a significant effect on the number of grains with perforations (*f*_0_ = 15.978, *p* < 0.01) and the number of grains with galleries (*f*_0_ = 22.318, *p* < 0.01), but not in the average weight of the perforated grains (*f*_0_ = 2.223, *p* = 0.145) (Table 3).

### 3.3. Losses Quantification

When accounting for losses, the two methods used to evaluate the efficacy of the tested compounds varied in the results regarding differences between treatments and blank control. Additionally, time had a significant effect on the production of maize powder and in *WGL* (*f*_0_ = 84.109, *p* < 0.01 and *f*_0_ = 15.968. *p* < 0.01, respectively), significantly increasing from OBS1 to OBS2 (Table 4). The determination of losses through the observation of the produced maize powder compared with the usage of *WGL* showed that the amount of maize powder produced was affected more by the treatments, with a significant reduction of up to 71% in maize powder in EP370 + AO. The same treatment also showed the best results in *WGL* with reductions of 47% relative to the blank control. Treatments P185, EP185, and EP370 were also able to significantly reduce the amount of maize powder produced relative to the blank control, with the efficacy augmenting with higher concentrations. P60 showed significantly less maize powder then E60. E60 and E60 + Adj showed no significant difference from the blank control nor any significant difference between them (Table 4).

### 3.4. Effects of Eugenol and Pulegone on Insect Activity

To understand the effects that eugenol and pulegone could have on the quality and quantity of damages produced per insect, a linear regression model was computed for blank control, E60, and P60. The slope of the line was analyzed to find the correlation between the total number of *S. zeamais* and maize powder, *WGL*, number of grains with perforations, and number of grains with galleries. It was observed that the computed linear regression model for treatment E60 did not significantly explain the relation between the total number of *S. zeamais* with the other variables. Thus, E60 with adjuvants (Adj) was used in this analysis since the model was accordant with the data and results from previous tests were very similar between these two treatments and thus could serve, with its due limitations, the comparison purpose of this analysis. The slope of the line describing the association between every variable and the total number of *S. zeamais* was the highest for eugenol treatments (E60 and E60 + Adj), and the lowest for the pulegone treatment, even when compared with the blank control. The regression line computed for every variable significantly explained the interaction with an increasing number of maize weevils for blank control, E60 + Adj, and P60, with the only exception being the regression computed for the number of grains with galleries for P60 treatment (Table 5).

## 4. Discussion

Regarding the toxicity of the tested compounds and their effects on population dynamics, when comparing eugenol vs. pulegone at the same concentrations, eugenol presented better results, with a significantly smaller population relative to control and a lower number of live maize weevils at the second observation (OBS2), but with no significant differences in mortality. It was noted, however, that the treatment with pulegone resulted in higher mortality at the first observation (OBS1), declining greatly with a longer period of storage. As such, the equal overall mortality observed between eugenol and pulegone may be in fact due to the higher vapor pressure of pulegone (0.123 mm Hg at 25 °C) [68] than eugenol (0.022 mm Hg at 25 °C) [36], thus explaining why pulegone was only more toxic in the shorter sampling observation (OBS1). The observation is concurrent with other authors that noted a low persistence of pulegone, affecting its bioactivity just after a few days [49]. Since eugenol is generally regarded as less toxic than pulegone, the significantly lower number of maize weevils present in the eugenol-treated containers may be due to an effect of the compound in the reproductive aspects of the insect. In fact, Ho et al. [69] observed a decrease in the fecundity of *S. zeamais* when treated with *Syzygium aromaticum* EO, which could explain the observed results. Additionally, eugenol has also been demonstrated to potentially have a detrimental effect on the development of immature stages (egg, larvae, and pupae) inside grains [34], thus enabling the observed suppression of the population of *S. zeamais*.

To the best of our knowledge, the effects of pulegone in the development time of *S. zeamais* have not yet been studied. However, understanding this possible effect may be relevant, since according to our results, containers treated with P60 showed a significantly higher total number of *S. zeamais* relative to the blank control. It could be hypothesized that the toxic effect of pulegone leads to a shorter developmental period and/or lifespan, hence the faster development and higher number of sampled individuals. These effects may also have translated into the type of damages observed since the pulegone treatment with the highest concentration had one of the lowest amounts of grains with galleries. It could be hypothesized that due to its effect in a shorter developmental period, this led to earlier hatching and thus the diminished number of grains with visible galleries, but such a conclusion requires further research. As expected, increasing the concentration of pulegone led to higher mortality. However, this increase was not sufficient to entirely counteract the higher vapor pressure of the compound, since its effect once again declined greatly when observed after 20 weeks (OBS2).

It was also noted that the number of alive *S. zeamais* may be dependent on the population density and food availability, since even the blank control saw a big increase in mortality in the second observation. This behavior has also been observed by Fragoso et al. [70], where mortality rapidly started increasing after 120 days of storage and was concurrent with the increase of population. Additionally, the recipients utilized to store the maize in this study were closed with a HDPE cap with a seal, which can also affect the survivability of maize weevils [71]. Given that the last sampling observation in this study was after 20 weeks, the observed mortality and low number of alive *S. zeamais* in the blank control may be explained by the same behavior.

The treatments that better controlled the population of *S. zeamais* were those of combined eugenol and pulegone at concentrations equal or higher than $185\;\mu L\cdot L_{\mathrm{air}}^{-1}$. Although the total number of *S. zeamais* was not significantly different relative to the blank control, overall, these treatments displayed over 50% more mortality relative to blank control, with over 80% less alive *S. zeamais*. The combination of various EOs in a mixture has proven to be beneficial in lowering the dosages needed for the control of *S. zeamais* in other studies, demonstrating the potential for synergistic effects [49]. While the synergistic effects of eugenol and pulegone have not yet been proven on *S. zeamais*, this combination of active compounds has shown synergistic effects in other arthropods [40]. Although eugenol and pulegone are two well-studied botanical insecticides present in plant extracts, with demonstrated toxicity against *S. zeamais*, the mechanisms of action behind their toxic effects are not the same. The toxicity of pulegone is mainly attributed to the formation of menthofuran, a highly toxic organic compound, when pulegone is absorbed and oxidized in cytochrome P450 [46,47]. Eugenol can target octopamine receptors, acting as its homologous, thus inducing hyperactivation of the insect [72]. Eugenol can also inhibit acetylcholinesterase, thus disturbing nerve impulse transmission, as shown in *Sitophilus oryzae* [73]. Since the ability to target different sites within the insect’s nervous system is one of the key aspects in augmenting the toxicity of a given insecticide in a mixture [42], there is the possibility that eugenol and pulegone are also synergistic in their activity on *S. zeamais.* Another advantage in utilizing various EO active compounds in a mixture lies in the reduced risk of enabling the onset of resistant populations [74], a challenge that is often presented when designing control strategies for the maize weevil [19,75,76]. Further studies on the synergetic effects of these compounds to control *S. zeamais* are recommended.

In this study, two different methods were used and compared to assess losses. The Weight Grain Loss method, also known as the gravimetric method, is one of the standard procedures used to determine grain losses due to its practicality and ease of use. However, the application of this method and results obtained from its application can be limited when the size and weight of the maize kernels is not uniform, thereby limiting the conclusions that could be drawn.

The other method utilized in this work was the quantification of losses through the measurement of the produced maize powder. Although not standard, material collected in the form of powder in a closed container during the storage of maize can be attributed to the activity, feeding, and development of the insect. However, this method also has its drawbacks since the powder can not only be composed of maize leftovers from insect feeding, but also from frass and exoskeletal remains from insect development. Thus, this method may be too sensitive and results must be taken with prudence. Nevertheless, a clearer picture of the losses and their differences between treatments can be taken when looking at the results of both methods simultaneously.

The results have shown that the treatment that better restrained losses during the 20 weeks of storage was the combination of eugenol with pulegone at $370\;\mu L\cdot L_{\mathrm{air}}^{-1}$ plus antioxidant (AO). This treatment was able to reduce the amount of maize powder produced by 70% and the *WGL* by 46% relative to blank control, demonstrating the efficacy in combining both active compounds with this controlled release device.

EOs and its active compounds can also have an effect on food consumption, biomass gain rate, nutritional intake, and the efficiency of the conversion of ingested food in *S. zeamais* [24,77]. In this study, the total number of *S. zeamais* was the sampled group of *S. zeamais* that was better correlated with loss quantification (both maize powder and *WGL*) as well as two of the variables that could describe the observable damages (number of grains with perforations and number of grains with galleries). These results are in accordance with the characteristics of the life cycle of *S. zeamais* as well as their biology and feeding behavior. In fact, the higher correlation of the total number of *S. zeamais* rather than the number of alive *S. zeamais* can partially be a result of the sampling periods utilized in this study (after 10 weeks and after 20 weeks), since in favorable conditions, *S. zeamais’* typical life cycle in maize lasts for 34.7 days from egg to adult and they can live for up to 126 days [78]. During the assay, weevils that may have perished before each observation period would already have completed their development, perforating, feeding, and damaging the grain and producing maize powder. However, the increasing total number of *S. zeamais* was not correlated with the number of fragmented grains nor the number of fragments present in each of the collected samples. These two variables were hypothesized thus to not be exclusively a direct consequence of the activity of the insects, but also a consequence of mechanical damages as a result of the sieving process during maize preparation for the experiments, as well as the sieving process during sampling.

To establish whether the maize weevils were inflicting more damages per insect, the slope of the linear regression between the total number of *S. zeamais* and the variables selected for damages and losses was observed. In this study, it was noted that the slope of the line for eugenol-treated recipients was higher compared with the blank control, especially for the produced maize grain powder and *WGL*, while, inversely, pulegone showed a notably lower slope. The results from this analysis suggest that both pulegone and eugenol may have affected the food consumption of *S. zeamais*, with pulegone diminishing the number of losses per insect, while eugenol had the inverse effect. In our study, we observed that eugenol may be having an effect on the increased food consumption per insect, while other studies have observed the inverse effect, with eugenol significantly reducing the food consumption of *S. zeamais* adults [35]. However, this behavior was observed when applying eugenol in a food medium and at a concentration of $13.2\;\mathrm{mg}\cdot g_{\mathrm{food}}^{-1}$, a quantity severely higher than the one utilized in our study. Since the activity of EOs is closely bonded with the applied concentrations, the differences between the utilized concentrations may explain the different outcomes. Pulegone has also been observed to influence feeding behavior at the preingestional, ingestional, and postingestional phases of feeding [79]. Since the *R*^2^ values may not be high enough to draw a factual conclusion on the effects of these compounds on the number of inflicted damages per insect, the observed differences in slope only provide an outline on these possible effects and needs further research. As such, future investigation should focus on this matter.

## 5. Conclusions

Other works have followed the effects of EOs in maize storage and demonstrated its potential. However, the efficacy of these compounds was severely hampered by the need to apply these substances repeatedly and periodically in order to maintain their effects. This study demonstrated the potential application of Clove bud and Pennyroyal EOs in maize storage as well as their effects on maize weevil populations and the damages and losses associated with their activity. The device implemented in this study was a promising tool in the development of novel EO-based strategies to diminish losses in storage maize during long periods of storage. Through the usage of a Clove bud and Pennyroyal blend incorporated in a controlled release device, losses of maize were controlled by more than 45% over a long storage period of five months, profoundly diminishing the survivability of maize weevils by over 90%. The usage of the blend at a concentration of $370\;\mu L\cdot L_{\mathrm{air}}^{-1}$ with antioxidant (AO) showed the best results. Halving the concentration $\left(185\;\mu L\cdot L_{\mathrm{air}}^{-1}\right)$ still achieved a significant control of *S. zeamais* populations. Future work should be handled in exploring the effects of these compounds on progeny, fecundity, insect development, and feeding, since weevil mortality alone did not effectively determine a clear control of maize losses.

## Figures and Tables

**Table 1:** Mean (*n* = 5) total number of *S. zeamais* adults (*S. zeamais* Total), number of live *S. zeamais* (*S. zeamais* Alive) and mortality (*S. zeamais* Mortality (%)), for each treatment at the first (OBS1) and second (OBS2) observations, and relative to the blank control.

Treatment	*S. zeamais* Total			*S. zeamais* Alive			*S. zeamais* Mortality (%)		
	OBS1 *	OBS2 *	Relative **	OBS1 *	OBS2 *	Relative **	OBS1 *	OBS2 *	Relative **
B	74.37 ^ab^	83.32 ^a^	0.00 ^a^	44.60 ^d^	2.40 ^a^	0.00 ^cd^	27.96 ^a^	96.81 ^bc^	0.00 ^a^
E60	76.37 ^ab^	80.14 ^a^	−0.75 ^a^	33.20 ^cd^	1.40 ^a^	−26.38 ^bcd^	49.54 ^ab^	97.60 ^bc^	17.93 ^ab^
+Adj	67.78 ^a^	77.37 ^a^	−7.95 ^a^	26.60 ^abcd^	2.60 ^a^	−37.87 ^abc^	49.59 ^ab^	96.65 ^bc^	17.21 ^ab^
P60	96.59 ^b^	153.10 ^ab^	58.34 ^b^	30.80 ^bcd^	31.60 ^b^	32.77 ^d^	62.67 ^bc^	76.11 ^a^	11.22 ^ab^
P185	98.14 ^b^	115.14 ^ab^	35.25 ^ab^	4.80 ^a^	17.00 ^ab^	−53.62 ^abc^	93.28 ^d^	86.78 ^ab^	44.31 ^cd^
EP164	81.83 ^ab^	178.76 ^b^	65.25 ^b^	9.20 ^abc^	26.40 ^b^	−24.26 ^bcd^	84.84 ^cd^	80.58 ^a^	32.58 ^bc^
EP185	82.98 ^ab^	102.97 ^ab^	17.92 ^ab^	7.00 ^ab^	1.80 ^a^	−81.28 ^ab^	89.54 ^cd^	97.89 ^bc^	50.22 ^cd^
EP370	97.60 ^b^	78.52 ^a^	11.68 ^ab^	1.60 ^a^	0.00 ^a^	−96.60 ^a^	97.98 ^d^	100.00 ^c^	58.67 ^d^
+AO	75.74 ^ab^	73.97 ^a^	−5.06 ^a^	3.00 ^a^	0.00 ^a^	−93.62 ^ab^	95.21 ^d^	100.00 ^c^	56.45 ^cd^
Time **	Wilks’ Lambda = 0.739 $F_{0}\sim F\left(1,36\right),f_{0}=12.720,p<0.01$			Wilks’ Lambda = 0.742 $F_{0}\sim F\left(1,36\right),f_{0}=12.535,p<0.01$			Wilks’ Lambda = 0.353 $F_{0}\sim F\left(1,36\right),f_{0}=65.905,p<0.01$		
Time × Treatment **	Wilks’ Lambda = 0.543 $F_{0}\sim F\left(8,36\right),f_{0}=3.785,p<0.01$			Wilks’ Lambda = 0.376 $F_{0}\sim F\left(8,36\right),f_{0}=7.454,p<0.01$			Wilks’ Lambda = 0.253 $F_{0}\sim F\left(8,36\right),f_{0}=13.302,p<0.01$		

Mean values followed by different lowercase letters for statistical difference at 95% confidence level: *p* < 0.05. Lowercase letters were determined by: *: One-way ANOVA with a Tukey HSD test performed independently for each observation. **: Repeated measures one-way ANOVA with a post-hoc Tukey HSD test performed relative to the blank control: values presented in percentage of reduction (negative) or increase (positive).

**Table 2:** Bivariate correlation analysis (Pearson’s *r* value) on two sampled groups of *S. zeamais* correlating damages observed in the maize grains. *N* = 90 for all computed variables.

	Maize Powder	*WGL*	PerfG	ChipG	GGall	GFrag
*S. zeamais* Total	0.361 **	0.688 **	0.686 **	−0.009	0.356 **	−0.021
*S. zeamais* Alive	0.051	0.232 *	0.235 *	0.135	−0.060	0.057

Significant correlations are indicated as: * for *p* < 0.05; ** for *p* < 0.01.

**Table 3:** Mean (*n* = 5) number of maize grains collected with observable damages, separated by type, for each treated container at the first (OBS1) and second (OBS2) observations, relative to the blank control.

Treatment	PerfG			AWPerfG			GGall		
	OBS1 *	OBS2 *	Relative **	OBS1 *	OBS2 *	Relative **	OBS1 *	OBS2 *	Relative **
B	14.00 ^a^	25.00 ^a^	0.00 ^ab^	0.29 ^a^	0.28 ^a^	0.00 ^a^	3.60 ^a^	13.00 ^a^	0.00 ^a^
E60	12.40 ^a^	17.20 ^a^	−24.10 ^ab^	0.30 ^a^	0.29 ^ab^	3.51 ^a^	2.00 ^a^	14.80 ^a^	1.20 ^a^
+Adj	9.60 ^a^	17.60 ^a^	−30.26 ^ab^	0.32 ^a^	0.28 ^a^	5.26 ^a^	3.00 ^a^	12.20 ^a^	−8.43 ^a^
P60	13.20 ^a^	27.20 ^a^	3.59 ^ab^	0.30 ^a^	0.29 ^ab^	3.16 ^a^	3.40 ^a^	4.60 ^a^	−51.81 ^a^
P185	15.20 ^a^	18.40 ^a^	−13.85 ^ab^	0.29 ^a^	0.31 ^ab^	5.61 ^a^	3.40 ^a^	3.00 ^a^	−61.45 ^a^
EP164	13.00 ^a^	27.80 ^a^	4.62 ^b^	0.31 ^a^	0.30 ^ab^	6.32 ^a^	1.80 ^a^	10.20 ^a^	−27.71 ^a^
EP185	14.60 ^a^	20.80 ^a^	−9.23 ^ab^	0.29 ^a^	0.29 ^ab^	1.75 ^a^	1.80 ^a^	7.60 ^a^	−43.37 ^a^
EP370	14.20 ^a^	15.00 ^a^	−25.13 ^ab^	0.30 ^a^	0.30 ^ab^	5.26 ^a^	1.40 ^a^	5.80 ^a^	−56.63 ^a^
+AO	11.80 ^a^	9.60 ^a^	−45.13 ^a^	0.31 ^a^	0.32 ^b^	10.18 ^a^	1.80 ^a^	2.20 ^a^	−75.90 ^a^
Time **	Wilks’ Lambda = 0.693 $F_{0}\sim F\left(1,36\right),f_{0}=15.978,p<0.01$			Wilks’ Lambda = 0.942 $F_{0}\sim F\left(1,36\right),f_{0}=2.223,p=0.145$			Wilks’ Lambda = 0.617 $F_{0}\sim F\left(1,36\right),f_{0}=22.318,p<0.01$		
Time × Treatment **	Wilks’ Lambda = 0.774 $F_{0}\sim F\left(8,36\right),f_{0}=1.319,p=0.266$			Wilks’ Lambda = 0.458 $F_{0}\sim F\left(8,36\right),f_{0}=1.436,p=0.216$			Wilks’ Lambda = 0.733 $F_{0}\sim F\left(8,36\right),f_{0}=1.637,p=0.149$		

Mean values followed by different lowercase letters for statistical difference at 95% confidence level: *p* < 0.05. Lowercase letters were determined by: *: One-way ANOVA with a Tukey HSD test performed independently for each observation. **: Repeated measures one-way ANOVA with a post-hoc Tukey HSD test performed relative to the blank control: values presented in percentage of reduction (negative) or increase (positive).

**Table 4:** Mean (*n* = 5) weight of collected maize powder and weight grain loss (*WGL*) values at the first (OBS1) and second (OBS2) observations, relative to the blank control, for each treated container.

Treatment	Maize Powder (g.kg^−1^)			*WGL*		
	OBS1 *	OBS2 *	Relative **	OBS1 *	OBS2 *	Relative **
B	0.62 ^bc^	2.01 ^b^	0.00 ^bc^	2.34 ^a^	4.22 ^a^	0.00 ^ab^
E60	0.80 ^c^	2.08 ^b^	9.11 ^c^	2.05 ^a^	2.87 ^a^	−24.94 ^ab^
+Adj	0.49 ^abc^	2.02 ^b^	−4.71 ^bc^	1.58 ^a^	2.95 ^a^	−30.91 ^ab^
P60	0.45 ^ab^	1.37 ^ab^	−30.90 ^abc^	2.19 ^a^	4.49 ^a^	1.95 ^ab^
P185	0.25 ^a^	0.90 ^ab^	−56.49 ^a^	2.51 ^a^	3.00 ^a^	−15.91 ^ab^
EP164	0.22 ^a^	1.25 ^ab^	−43.96 ^ab^	2.27 ^a^	4.64 ^a^	5.27 ^b^
EP185	0.18 ^a^	0.73 ^a^	−65.30 ^a^	2.41 ^a^	3.43 ^a^	−11.07 ^ab^
EP370	0.27 ^a^	0.51 ^a^	−70.08 ^a^	2.33 ^a^	2.48 ^a^	−26.71 ^ab^
+AO	0.25 ^a^	0.52 ^a^	−70.62 ^a^	1.93 ^a^	1.58 ^a^	−46.55 ^a^
Time **	Wilks’ Lambda = 0.300 $F_{0}\sim F\left(1,36\right),f_{0}=84.109,p<0.01$			Wilks’ Lambda = 0.693 $F_{0}\sim F\left(1,36\right),f_{0}=15.968,p<0.01$		
Time × Treatment **	Wilks’ Lambda = 0.620 $F_{0}\sim F\left(8,36\right),f_{0}=2.756,p<0.05$			Wilks’ Lambda = 0.779 $F_{0}\sim F\left(8,36\right),f_{0}=1.276,p=0.287$		

Mean values followed by different lowercase letters for statistical difference at 95% confidence level: *p* < 0.05. Lowercase letters were determined by: *: One-way ANOVA with a Tukey HSD test performed independently for each observation. **: Repeated measures one-way ANOVA with a post-hoc Tukey HSD test performed relative to the blank control: values presented in percentage of reduction (negative) or increase (positive).

**Table 5:** Line slope (*B*) of the linear regression (*n* = 10) calculated for the variation of maize powder, *WGL*, PerfG, and GGall with the total number of *S. zeamais* per container. Regression analysis made independently for each treatment and blank control.

Treatment	Maize Powder				*WGL*				PerfG				GGall			
	*B* ± SE	*R* ^2^	*t*	*p*-Value	*B* ± SE	*R* ^2^	*t*	*p*-Value	*B* ± SE	*R* ^2^	*t*	*p*-Value	*B* ± SE	*R* ^2^	*t*	*p*-Value
B	0.027 ± 0.008	0.581	3.329	0.01	0.069 ± 0.010	0.863	7.108	0.00	0.393 ± 0.059	0.849	6.710	0.00	0.240 ± 0.075	0.560	3.189	0.01
E60	0.047 ± 0.034	0.188	1.359	0.21	0.063 ± 0.037	0.267	1.708	0.13	0.354 ± 0.225	0.237	1.575	0.15	0.732 ± 0.416	0.279	1.757	0.12
+Adj	0.066 ± 0.022	0.524	2.968	0.02	0.105 ± 0.025	0.683	4.155	0.00	0.627 ± 0.147	0.695	4.274	0.00	0.555 ± 0.165	0.586	3.365	0.01
P60	0.014 ± 0.003	0.666	3.997	0.00	0.040 ± 0.007	0.805	5.752	0.00	0.240 ± 0.042	0.898	5.765	0.00	0.038 ± 0.019	0.346	2.059	0.07

Statistical significance of linear regression model: *p* < 0.05. *t*—test statistic observed value. *R*^2^—determination coefficient.

## Data Availability

The data presented in this study are available upon request from the corresponding author. The data are not publicly available due to the uncompleted subject.
